# Role of Pore-Forming Toxins in Neonatal Sepsis

**DOI:** 10.1155/2013/608456

**Published:** 2013-04-23

**Authors:** Andreas F.-P. Sonnen, Philipp Henneke

**Affiliations:** Center of Chronic Immunodeficiency, Medical Center, University of Freiburg, Breisacher Straße 117, 79106 Freiburg, Germany

## Abstract

Protein toxins are important virulence factors contributing to neonatal sepsis. The major pathogens of neonatal sepsis, group B Streptococci, *Escherichia coli*, *Listeria monocytogenes*, and *Staphylococcus aureus*, secrete toxins of different molecular nature, which are key for defining the disease. Amongst these toxins are pore-forming exotoxins that are expressed as soluble monomers prior to engagement of the target cell membrane with subsequent formation of an aqueous membrane pore. Membrane pore formation is not only a means for immediate lysis of the targeted cell but also a general mechanism that contributes to penetration of epithelial barriers and evasion of the immune system, thus creating survival niches for the pathogens. Pore-forming toxins, however, can also contribute to the induction of inflammation and hence to the manifestation of sepsis. Clearly, pore-forming toxins are not the sole factors that drive sepsis progression, but they often act in concert with other bacterial effectors, especially in the initial stages of neonatal sepsis manifestation.

## 1. Introduction

The birth canal, that is, the area between the fully dilated uterus and the outside of the vagina, harbours a polymicrobial community that may fulfil the definition of a biofilm [1]. Next to apathogenic species, such as *Lactobacillus *spp., potentially pathogenic bacteria including group B streptococci (GBS), *Escherichia coli* (*E. coli*), *Listeria monocytogenes* (*L. monocytogenes*), and *Staphylococcus aureus* (*S. aureus*) are found in the vagina of up to 20% of women. During birth, the fetus needs to pass from the sterile uterus through these bacteria. Accordingly, aspiration of bacteria during birth is regarded as a major cause of neonatal sepsis in the first three to seven days of life (early-onset sepsis). In line with this model, early-onset sepsis is predominantly caused by GBS, *E. coli*, *L. monocytogenes*, and *S. aureus*. Yet most infants successfully control the bacteria at the mucocutaneous surfaces. 

Subsequent to aspiration, bacteria like *GBS* can proliferate to striking densities in the neonatal lung, as shown in newborn primates with neonatal GBS pneumonia (10^9^–10^11^ colony-forming units (CFUs)/g lung tissue, [2]). The antimicrobial quality of the local pulmonary environment, for example, the concentration of surfactant, may be important for the metabolic activity in the bacterial community and therefore for the expression of bacterial virulence factors such as bacterial toxins [3].

Sepsis imposes a major threat to newborn infants. It is estimated that sepsis causes over half a million neonatal deaths annually, thereby accounting for about 15% of all neonatal deaths worldwide [4]. Whereas sepsis causes approximately 2.5% of infant deaths in developed countries, it is responsible for up to 50% of neonatal deaths in developing countries [5, 6]. Moreover, neonatal sepsis often occurs as meningoencephalitis, which leaves almost 50% of affected patients with lifelong disabilities [7]. On the other hand, GBS, *E. coli*, and *S. aureus* are normal components of the mucocutaneous microbiome, and it is impossible to predict the risk to an individual baby.

## 2. Bacterial Membrane-Damaging Toxins 

**Figure 1:**
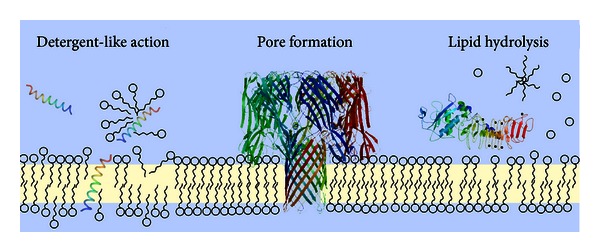
Ways to damage a lipid membrane. There are various mechanisms of membrane damage by protein toxins. Amphiphilic toxins can integrate into the membrane and essentially solubilise the lipid membrane like a detergent (structure: *S. aureus δ*-toxin, PDB ID 2KAM). Similarly, the membrane lipids can be hydrolysed by phospholipases also resulting in the destruction of the membrane (structure: *Clostridium, perfringens α*-toxin, PDB ID 1KHO, [165]). By far the largest class of membrane damaging toxins is that of the pore-forming toxins (structure: *α*-toxin from *S. aureus*, PDB ID 7AHL, [100]). These toxins integrate as stable channels into the lipid bilayer, thus creating an aqueous connection between the cytosol and the extracellular space of the target cell.

The first membrane-damaging bacterial toxin was described by Paul Ehrlich in 1898 [8], who found that *Clostridium tetani* extracts lyse erythrocytes. Today, three different mechanisms of membrane damage by proteinaceous agents can be delineated. First, toxins can solubilise target membranes acting essentially as amphiphilic surfactants. *δ*-Toxins from various staphylococcal species [9, 10] and the cyclolipopeptides from *Bacillus subtilis* are prominent examples [11] (see Figure 1). Second, toxins can act as phospholipases and damage membranes by enzymatic hydrolysis of phospholipid ester bonds. *β*-Hemolysin from *S. aureus*, for instance, is a sphingomyelin-specific phospholipase, which cleaves sphingomyelin to ceramide and phosphorylcholine. However, the large majority of membrane damaging proteins belong to the class of pore-forming proteins/toxins (PFTs). PFTs, which make up approximately 30% of all protein toxins in pathogenic bacteria [12], have evolved in all domains of life. They are secreted as water-soluble proteins and subsequently integrate into foreign membranes. 

## 3. Mechanism of Membrane Pore Formation by Pore-Forming Toxins

**Figure 2:**
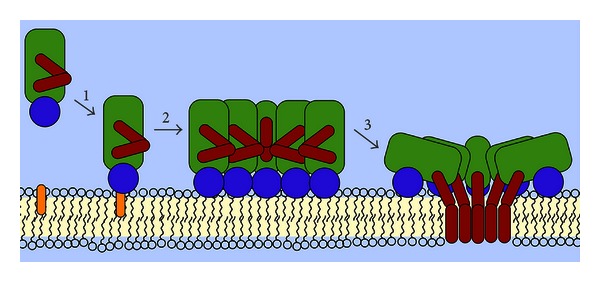
Pore formation is a dynamic process with structurally and functionally distinct states. Distinct molecular states exist on the path to membrane pore formation by PFTs. The toxin is secreted by the bacterial pathogen into the extracellular medium in a water-soluble form, usually as a monomer. Upon engagement of the membrane via binding to a receptor (step 1), for example, a membrane lipid or protein, the monomers assemble to a prepore oligomer (step 2). The membrane beneath the prepore oligomer remains intact and is only punctured once the prepore refolds to the membrane-inserted pore oligomer (step 3). This step usually goes along with considerable structural rearrangements.

**Figure 3:**
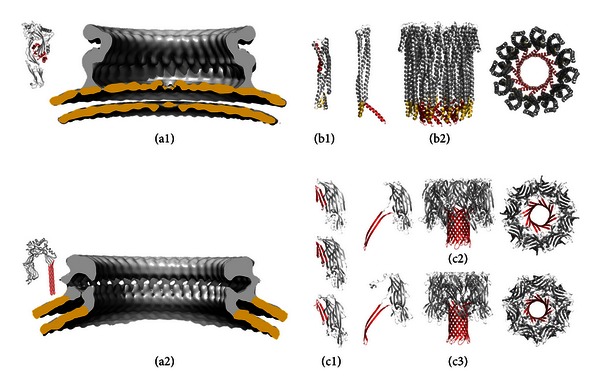
Structures of PFTs that are important for neonatal sepsis. Panel (a) shows available structures of cholesterol-dependent cytolysins to illustrate listeriolysins' mechanism of pore formation. (a1) displays the crystal structure of the soluble, monomeric form of perfringolysin from *Clostridium perfringens* (left, PDB ID 1PFO, [57]). The cryo-electron microscopy (cryo-EM) reconstruction of the prepore (EM databank: 1106) of the listeriolysin homologue pneumolysin from *Streptococcus pneumoniae* displayed on the right revealed that the protomer configuration in the prepore resembles that of the soluble monomer [16]. Lipid membrane is coloured yellow. Molecular modeling of the protomer fitted into the cryo-EM pore structure below (EM databank: 1107) revealed the considerable structural rearrangements that accompany membrane pore formation. The *α*-helices that refold into *β*-sheets are coloured in red. Panel (b) shows the different structures available for ClyA from *E. coli*, (b1) the soluble state (PDDid 1QOY, [148]) monomer and (b2) a protomer from the dodecameric pore state, which is shown as side and top view on the right (PDB ID 2WCD, [149]). Pore-lining *α*-helices are in red and the *β*-tongue in yellow. Panel (c) shows the PFTs from *S. aureus*. (c1) shows from top to bottom LukF (PDB ID 1LKF, [113]), LukF-PV (PDB ID 1PVL, [114]), and LukS-PV (PDB ID 1T5R, [115]). (c2) shows the octameric pore structure of *γ*-hemolysin (PDB ID 3B07, [116]), protomer on the left, side and top views on the right. (c3) displays the heptameric pore structure of the AFT pore (PDB ID 7AHL, [100]), individual protomer, side and tops views. The *β*-stem that unfolds into the membrane lining, extended *β*-hairpin is shown in red.

Common structural themes of protein/membrane association are insertion of transmembrane *α*-helices or *β*-sheet barrel arrangements, anchoring by prosthetic glycolipids, or direct linkage to hydrophobic lipid tails, such as myristic or palmitic acid. Most pores or channels allowing for communication across biological membranes are formed by integral membrane proteins spanning the lipid bilayer. However, PFTs form pores by acting initially extraneously of the lipid bilayer. They start out as soluble molecules and then turn themselves into integral membrane proteins, with a membrane-spanning region that defines the pore. Pore formation is a dynamic process with structurally and functionally distinct states (see Figure 2). Initial binding to the membrane, for example, to a membrane lipid or protein receptor, is followed by homotypic oligomerisation to a prepore state on the membrane surface (Figure 2, arrows 1 and 2). In this state, the protomer configuration resembles that of the soluble monomer, and the whole oligomer still stands prone to the membrane with an intact lipid bilayer beneath the assembled ring. In Figure 3(a1), this is depicted for pneumolysin from *Streptococcus pneumoniae*, a close homologue of listeriolysin from *L. monocytogenes* and, interestingly, also of perforin secreted by cytotoxic T cells [13], and of the complement membrane attack complex [14, 15], which indicates that bacterial attack and immune defence employ the same mechanisms. This prepore state then undergoes drastic conformational rearrangements to be inserted as a stable pore into the membrane (see Figure 2, arrow 3). This rearrangement can even involve the refolding of *α*-helices in the soluble state to *β*-sheets in the membrane-inserted form [16]. While this general mechanism of pore formation can be proposed for nearly all PFTs, the structural changes, which the individual proteins undergo, remain largely elusive. Detailed mechanistic models on how hydrophilic proteins can suddenly change their solubility and integrate into biological membranes are available only for a few PFTs involved in neonatal sepsis (see Figure 3). Not surprising when considering that one not only needs to be able to study the solution state in sufficient structural detail, for example, via X-ray crystallography, but the structure of the membrane state also needs to be resolved. PFTs are classically divided into two main groups based on the structural motifs that form the pore [17–19]. Pores can be formed by *α*-helices, *α*-PFTs or by *β*-sheets, *β*-PFTs. For certain pore-forming proteins, it was recently proposed that lipids might play a direct role in pore formation, but our understanding of how this can be achieved is limited by the available structural data to date [20–22]. The structures, where known, of the soluble and membrane states of the PFTs discussed in this review are displayed in Figure 3. However, not all membrane pores are equal. The pore diameter, for instance, of the *α*-hemolysin membrane heptamer is considerably smaller than that of listeriolysin with up to 50 protomers (cf. Figures 3(a) and 3(c)). Clearly, the size of the membrane pore has important consequences for the targeted cell, as a large pore diameter is not selective for what it can conduct across a membrane, potentially mediating diffusion of larger molecules, such as ATP or even small proteins. 

Interestingly, clearing of toxin pores from host membranes, a mechanism that is of marked importance when considering the amounts of toxin that are produced during sepsis, seems also to be size dependent. Cholesterol-dependent cytolysins, such as listeriolysin, can induce Ca^2+^-dependent resealing of membrane pores by induction of endocytosis [23–25] but *α*-hemolysin from *S. aureus* cannot [26]. This is counterintuitive, as van der Goot and coworkers nicely state [27] that small pores are harder to repair than larger ones.

## 4. Bacterial Pore-Forming Toxins in Pathogens Causing Neonatal Sepsis

In the major pathogens isolated from newborn infants with sepsis, PFTs are key virulence factors. They initiate a multitude of events ranging from direct necrotic cell deaths to the induction of signalling cascades, for instance, Ca^2+^-mediated signalling [27]. Prominent PFTs in the context of neonatal sepsis are listeriolysin O from *L. monocytogenes*, *β*-hemolysin/cytolysin from GBS (*Streptococcus agalactiae*), *α*-hemolysin and cytolysin A from *E. coli*, and *α*-hemolysin, *γ*-hemolysin, and the leukocidins from *S. aureus*. It has long been appreciated that PFTs are especially important during initiation of bacterial infections through induction of necrosis and apoptosis of host epithelial and endothelial cells, which promotes microbial invasion and subverts defence mechanisms. However, PFTs may also contribute to sepsis by receptor mediated or membrane damaging mechanisms in immune cells, which respond with the formation of inflammatory mediators, as was shown for *β*-hemolysin/cytolysin from GBS [28] and listeriolysin O [29, 30] and *α*-hemolysin and Panton-Valentine leukocidins from *S. aureus* [31, 32].

## 5. ***β***-Hemolysin/Cytolysin from Group B Streptococci

*Streptococcus agalactiae* (group B streptococci, GBS) are the major cause of sepsis and meningitis in newborn infants without underlying disease in the western world. In addition, they are a significant cause of invasive infections in pregnant woman and immuneocompromised patients [33, 34]. The pore-forming toxin *β*-hemolysin/cytolysin is one of the main virulence factors of GBS. It has been implicated in the pathogenesis of early- [35] and late-onset neonatal sepsis, although its role in both cases remains controversial. Rabbits infected with wild-type GBS had significantly higher bacterial blood counts than those infected with GBS mutants lacking the *β*-hemolysin/cytolysin [35]; mortality also increased dramatically. Similarly, in a neonatal rat model of meningitis wild-type GBS induced more neuronal damage in the cortex and the hippocampus than cytolysin-deficient mutants [36]. The clinical outcome score, assessed in this study by weighted changes and motor activity, decreased profoundly upon presence of the cytolysin. The *β*-hemolysin/cytolysin lytic protein agent is thought to be encoded by the cylE gene of the cyl operon [37], since expression of cylE induces *β*-hemolytic activity in nonhemolytic *E. coli*. However, the exact molecular nature of the protein component responsible for hemolysis and pore formation remains obscure, as it has evaded purification to homogeneity as of yet. Interestingly, *β*-hemolysin/cytolysin is also necessary for the synthesis of an orange carotenoid pigment [38], with which it also associates [39]. Hemolytic activity of partially purified *β*-hemolytic activity containing the carotenoid pigment could be inhibited by addition of the lipid dipalmitoylphosphatidylcholine (DPPC), the major component of surfactant in the lung. Accordingly, surfactant deficiency may explain in part the particular susceptibility of preterm infants to GBS sepsis and meningitis [40, 41]. As outlined above, breaching of epithelial barriers by GBS is the first step in sepsis pathogenesis. Accordingly, it appears to be important that *β*-hemolysin/cytolysin mediates not only injury of lung epithelial [40] and of lung microvascular endothelial cells [42] and invasion of brain endothelial cells [43] but also injury of professional phagocytes [39] and neurons [36]. Moreover, GBS mutants lacking clyE were more readily cleared from mouse and human blood [39]. Interestingly, Rubens and colleagues initially proposed that *β*-hemolysin/cytolysin was no longer needed for systemic disease manifestation once the epithelial barriers have been breached [44]. However, pro- and anti-inflammatory activity of *β*-hemolysin/cytolysin in macrophages was demonstrated, indicating more profound immunomodulatory functions of the cytolysin [45, 46]. One direct or indirect molecular *β*-hemolysin/cytolysin target with important implications for mononuclear phagocyte activation is the NLPRP3 inflammasome. Activation of the NLRP3 inflammasome requires GBS expressing *β*-hemolysin/cytolysin. This pathway is essential in a mouse model of GBS sepsis, where deficiency in NLRP3 or its signalling partners apoptosis-associated speck-like protein and caspase-1 increases lethality and bacterial dissemination [28]. Yet direct evidence for binding, engagement, and activation of TLRs by the *β*-hemolysin/cytolysin is not available.

A second pore-forming toxin of GBS is the CAMP factor that has long been used for microbiological identification of GBS, since it characteristically synergizes with secreted sphingomyelinase of *S. aureus* to lyse erythrocytes on blood agar plates [47–49]. However, its role in neonatal sepsis is not clear, as it was not required for systemic infection in a mouse model of GBS infection [50].

## 6. Listeriolysin O from *Listeria monocytogenes*

*Listeria monocytogenes* (*L. monocytogenes*) is a Gram-positive bacterium that causes early- and late-onset neonatal sepsis and meningitis. *L. monocytogenes* has the capacity to breach the intestinal barrier, thereby causing food-borne listeriosis, the blood-brain barrier, causing meningitis, and the maternal-placental barrier, causing early-onset listeriosis. Listeriolysin O (LLO), a member of the PFT class of cholesterol-dependent cytolysins (CDCs), is a major virulence factor of *L. monocytogenes* with multivalent functions [51]. In the late 1980s, Kathariou et al. and Portnoy et al. reported that *L. monocytogenes* mutants lacking functional LLO were avirulent in mice [52, 53]. Furthermore, LLO mutants did not induce secretion of cytokines such as TNF-*α*, IL-1*β*, or IFN-*γ*, when injected intravenously into C57BL/6 mice [54]. Recently, a single-gene signature-tag-based approach was used to assess the contribution of individual amino acids of LLOs to its virulence in mice [55]. 

 Based on structural homology with other toxins such as pneumolysin from *Streptococcus pneumoniae* [56] and perfringolysin from *Clostridium perfringens* [57], common pore-forming properties can be proposed [58] (see Figure 3(a)). LLO engages cholesterol as a native membrane receptor in dependence on the two amino acids threonine 515 and leucine 516, oligomerises to a prepore complex of up to 50 monomers, and forms a membrane pore in a concerted refolding step with each protomer contributing two beta-hairpins to the membrane-spanning *β*-barrel, which originates from five *α*-helices in the soluble state [16, 59–61]. The allosteric monomer assembly and prepore refolding process were recently shown to rely on an undecapeptide sequence (483-ECTGLAWEWWR-493), which was originally thought to be soley responsible for cholesterol binding [62]. The exact nature of the membrane pore remains controversial, as arciform pores, that is, membrane pores with a seemingly incomplete protein ring lining the aqueous membrane hole, are often observed in electron microscopic and atomic force microscopic imaging of various CDCs [22, 63–65]. Moreover, LLO does not lose its membrane targeting properties after incubation with cholesterol [66].

*Listeria* are classical intracellular pathogens [67] and LLO pore formation was traditionally thought to only mediate escape of *Listeria* from the phagolysosome [68]. This concept was based on the finding that LLO was active only at acidic and not at neutral pH, which is found in the maturing phagolysosome [58]. However, host factors also play an important part in regulating the activity of LLO in the phagolysosome. LLO hijacks the reductive capacity of the *γ*-interferon-inducible lysosomal thiol reductase GILT to maintain cysteine 484 of LLO in its reduced thiol state [69], thus greatly increasing bacterial escape from the phagolysosome. Intriguingly, CDCs were originally, that is, before the identification of cholesterol as a membrane receptor, termed sulfhydryl-activated [70] or thiol-activated, oxygen-labile cytolysins [71], as chemical reduction activated the toxins towards hemolysis of red blood cells. Additionally, the cystic fibrosis transmembrane conductance regulator (CFTR), which transports chloride not only across the apical plasma membrane of epithelial cells in the lung but also into the phagolysosomes of macrophages, potentiates LLO oligomerisation on the phagosomal membrane and its lytic activity and phagolysosomal escape of *L. monocytogenes* [72]. However, the role of LLO extends beyond mediating phagosomal escape. LLO reduces formation of reactive oxygen species (ROS) by inhibiting the NADPH oxidase NOX2 in RAW 264.7 macrophages [73]. This activity seems to rely on pore formation in the phagosomal membrane and prevents degradation of bacteria inside the phagosome. Pore formation at the plasma membrane of target cells induces the dynamin-/F-actin-dependent but clathrin-independent uptake of *L. monocytogenes* into HepG2 cells [74]. This finding questions the traditional model of LLO pore-forming activity being strictly dependent on the low phagosomal pH [58], whereby premature lysis of target cells by (secreted) LLO is prevented [75]. Residual lytic activity and structural integrity of LLO at neutral pH [74, 76, 77] are in line with LLO-mediated calcium influx into epithelial Hep-2 [78] and HEK 293 cells [79] along with concomitant *L. monocytogenes* uptake. Indeed LLO seems to form pores at neutral pH in the plasma membrane, which do not result in lysis of the target cell but rather in uptake of the pathogen [74]. As pneumolysin from *S. pneumoniae* can replace LLO in the uptake of *L. monocytogenes* into HepG2 cells, CDCs from other bacterial pathogens may similarly induce cellular uptake. The contribution of TLR signalling in response to CDC has been subject of several studies. As examples, the LLO homologues anthrolysin (*Bacillus anthracis*) and pneumolysin can signal via TLR4 [80, 81]. On the other hand, LLO induces an inflammatory cellular response in a TLR-independent fashion [82]. Moreover, LLO activity at the plasma membrane induces clustering of lipid rafts [83], suppression of antigen-induced T-cell activation [84], inflammasome activation, and histone H3 dephosphorylation [30], all of which might contribute to sepsis progression either at the stage of heightened inflammation or at later stages of immune suppression. 

## 7. ***α***-Hemolysin, ***γ***-Hemolysin, and Leukocidins from *Staphylococcus aureus*

*Staphylococcus aureus* (*S. aureus*) is well recognised as a significant cause of neonatal sepsis [85]. Around ten bacteria are sufficient to colonise the umbilical cord. After birth, *S. aureus* can colonise the upper respiratory tract in up to 40% of infants [86]. *S. aureus* produces a number of PFTs with distinct specificity for target cell membranes. Although most clinical isolates produce the PFTs *α*-hemolysin, bicomponent *γ*-hemolysins, and bicomponent leukocidins, none of these toxins was found to be a necessary and sufficient virulence determinant of neonatal sepsis. In contrast, other factors such as the antigenic, peptidoglycan-associated protein A [87], superantigens [88], and sphingomyelinase C (*β*-hemolysin, [89]) contribute to host invasion, subversion of the immune system, and sepsis manifestation. However, there is evidence that pore formation by *α*-hemolysin (*α*-toxin, AFT) contributes to the pathogenesis of sepsis [90]. As an example, erythrocyte lysis by AFT could be directly imaged [91]. Downregulation of AFT expression *in vivo* clearly reduces virulence of *S. aureus* [92, 93]. In a model using C57BL/6J mice, AFT activates the NLRP3 inflammasome, thereby promoting necrotising pneumonia [94]. Moreover, monoclonal antibodies to AFT are protective against staphylococcal pneumonia [95]. Indeed, a nonhemolytic variant of AFT was used to vaccinate rabbits, and antisera could be used to passively immunize mice against an otherwise lethal challenge with wild-type *S. aureus* [96]. In a mouse model of mastitis, coagulase and AFT proved to be the primary virulence determinants [97]. Heat-inactivated *S. aureus* and an AFT mutant greatly reduced the bacterial burdens in a mouse brain abscess model and attenuated the expression of inflammatory mediators [98]. In an *in vivo* model of corneal virulence, AFT also proved to be a decisive virulence factor [99]. 

AFT is one of the best-studied PFTs to date. It was the first toxin of which the membrane structure was solved by X-ray crystallography (see Figure 3(c3), [100]). AFT consists of 293 amino acids and oligomerises on the plasma membrane of target cells to a heptamer (potentially a hexamer) prior to membrane insertion and pore formation. It was shown to have an important role in bacterial pathogenesis, especially by its ability to induce necrotic cell death [90], by which it can cause vascular leakage when perfused into the lung [101]. Recently the metalloprotease ADAM10 was identified as the membrane receptor of AFT [102]. At low toxin concentrations, ADAM10 is required to mediate the cytotoxic effects of AFT. Interestingly, binding of AFT to ADAM10 resulted in the upregulation of ADAM10 in alveolar epithelial cells and concomitant cleavage of E-cadherin. This leads to epithelial barrier disruption thereby aggravating staphylococcal pneumonia in mice [103]. Moreover, activation of the NLRP3-inflammasome by AFT might contribute to later stages of sepsis, although the molecular mechanism underlying inflammasome activation remains elusive at this stage [31]. In this respect, it is interesting to note that, whereas direct TLR activation has not been demonstrated for AFT, NOD2-dependent sensing of *S. aureus* was dependent on AFT [104]. 

Next to AFT, *S. aureus* expresses the bicomponent cytotoxins leukocidins (Luk) and the *γ*-hemolysins (Hlg). Bicomponent implies that class S toxins (LukS-PV, LukE, HlgA, HlgC, and LukS) have to associate with class F toxins (LukF, LukD, LukF-PV, and HlgB) in a 1 : 1 stoichiometric ratio to form a functional oligomer before insertion into the membrane (see Figure 3(c)). Pathogenic *S. aureus* can produce several different bicomponent toxin pores, among the most prominent are LukE/LukD [105], Panton-Valentine leukocidin LukS-PV/LukF-PV, *γ*-hemolysins LukF/HlgA [106], HlgA/HlgB, HlgB/HlgC, and the M/F-PV-like leukocidins, all of which might be expressed at different stages during sepsis. The Panton-Valentine leukocidin (PVL), which was first isolated from furuncles in 1936 [107], is probably the most widely studied member. In a mouse model, secreted PVL promotes tissue invasion and causes necrotizing pneumonia, via mechanisms including upregulation of protein A and other adhesins [108]. PVL is an important factor in the early stages of skin infection, as shown in a rabbit skin infection model [109]. Association of *pvl* and *spa* (protein A) genes seems to be an important virulence determinant in methicillin-resistant *S. aureus* (MRSA). Moreover, PVL was reported to directly bind to TLR2 and induce inflammation in the mouse lung [110]. LukE/LukD promotes systemic bacterial growth *in vivo* by specifically targeting neutrophils [111]. Interestingly, via engagement of their native receptor CCR5 (CC-motif-chemokine-receptor type 5), LukE/LukD toxin pores clear antigen-presenting cells (macrophages and dendritic cells) and *S. aureus*-specific CCR5-positive Th1/Th17 cells [112], thus greatly contributing to the spread of *S. aureus* in the host. 

AFT, Hlg, and the leukocidins belong to the class of *β*-pore-forming toxins [17]. Despite their moderate sequence identity (around 30% for the pairwise alignment with AFT), they share a common structural fold (see Figure 3(c)). The structures of the soluble, monomeric LukF (Figure 3(c1), top, [113]), LukF-PV (Figure 3(c1), middle, [114]), LukS-PV (Figure 3(c1), bottom, [115]), and the octameric, membrane complex of Hlg (LukF/HlgA, Figure 3(c2), [116]) are available. A common molecular mechanism could be proposed in which a prestem, triple stranded *β*-sheet in the soluble monomer, refolds to a double, stranded, membrane-inserted *β*-sheet (Figures 3(c2) and 3(c3), left), resulting in a hexadeca- or tetradecastranded *β*-barrel that penetrates the membrane.

 The target membrane specificities of *S. aureus* PFTs hint towards their role in neonatal sepsis. Whereas AFT has a broad specificity and might thus be involved at the initial stages of sepsis manifestation, where the lung epithelium needs to be breached, bicomponent leukocidins and Hlgs mainly attack polymorphonuclear neutrophils, macrophages and lymphocytes [117, 118]. Bearing in mind that PVL does not attack lymphocytes and Hlg can be hemolytic, at least *in vitro*, their restricted but highly directed mode of attack predisposes the bicomponent leukocidins to be important factors for the subversion of the immune system once the initial barrier has been breached. 

## 8. ***α***-Hemolysin and Cytolysin A from *Escherichia coli*

Pathogenic *Escherichia coli* (*E. coli*) cause around 25% of invasive neonatal sepsis [119], and antibiotic resistance is an emerging threat in this context [120]. Generally, *E. coli* can persist in the intestine as a normal constituent of the intestinal microbiota. However, extraintestinal pathogenic *E. coli* (ExPECs) are the most common gram-negative bacterial species isolated from neonates with bacterial infections, and neonatal mortality from gram-negative sepsis remains high [121]. Urinary tract infections of pregnant women can lead to aspiration of ExPECs during partition with subsequent uncontrolled growth in the lung of the newborn and potential progression to a systemic infection. Pathogenic *E. coli* often secrete the pore-forming toxin *α*-hemolysin (HlyA, CylA), a 107 kDa member of the RTX class of toxins [122], which is usually associated with strains causing uropathogenic infections [123]. Deletion of HlyA in *E. coli* greatly reduced mortality and cytokine production as compared to the isogenic wild type bacteria in an intravenous infection model [124]. HlyA furthermore induced hemorrhagic bleeding of bladder tissue and exfoliation of urothelium when pathogenic bacteria were administered into the urethra [125]. HlyA is encoded by at least 50% of all ExPEC clinical isolates [126]. Around 80% of meningitis- and sepsis-associated *E. coli* belong to the K1 serotype [127, 128]. The *E. coli * K1 strain RS218 expressed HlyA in a zebrafish model of systemic infection [126] and the hlyA gene was present in more than 40% of *E. coli* from the genital tracts of pregnant women [129]. As a member of the repeats in toxin (RTX) family of hemolysins, the hlyA gene is part of the chromosomal hlyCABD operon, which also encodes a type 1 ABC transporter for secretion of the PFT. The amino acid toxin repeats, which are located in the C-terminal portion of the protein, are composed of the sequence GGXGCDXUX (with U being a large hydrophobic residue). These repeats are responsible for Ca^2+^ binding, which is a prerequisite for membrane association and pore formation by the N-terminal, hydrophobic, and acylated domain of HlyA [130]. Interestingly, other pore-forming proteins, such as the human cytotoxic lymphocyte encoded perforin, also require binding of Ca^2+^ for membrane association and pore formation [131]. HlyA oligomerises on the plasma membrane of target cells, where it accumulates in cholesterol-rich microdomains [132, 133]. However, rather than cholesterol being a direct, lipid membrane receptor as in the case of the CDCs listeriolysin or pneumolysin, cholesterol seems to contribute to the physicochemical environment necessary for HlyA membrane engagement. The exact nature of the pore formation mechanism is under considerable debate. It seems now accepted that HlyA forms membrane pores as an oligomer, at least in artificial membrane mimics [134–136], albeit possibly heterogeneous in size [137]. In this respect, it is intriguing that the P2X7 receptor and pannexin 1 were found to mediate HlyA-dependent pore formation [138, 139]. Sublytic doses of HlyA initiate degradation of paxillin and proteolytic cascades inside epithelial cells and macrophages, thus attenuating the inflammatory host response and promoting epithelial exfoliation [140]. Similarly, HlyA inhibits epithelial cytokine production potentially promoting epithelial invasion of *E. coli* [141]. 

Another PFT of *E. coli* is ClyA (also termed hemolysin-E or SheA), which is expressed by various pathogenic and nonpathogenic *E. coli* including K12 strains, that are also found in clinical isolates of neonatal meningitis [142], bacteremia [143, 144], and neonatal sepsis [145]. However, three neonatal meningitis K1 strains isolated by Ludwig and colleagues harboured deletion mutations at the clyA gene locus [146]. Nevertheless, a synergistic enhancement of extraintestinal infection was recently reported between nonpathogenic *E. coli* K12 and pathogenic ExPEC strains in a mouse model of septicaemia [147], which hints towards a potential involvement of ClyA. ClyA is a 34 kDa protein belonging to the class of *α*-PFTs. The mechanism of pore formation is well understood, as crystal structures for the soluble [148] and membrane state [149] are available (see Figure 3(b)). Upon association with the membrane, insertion of the so-called *β*-tongue induces a series of substantial structural rearrangements in the now membrane-anchored monomer, resulting in a perpendicular position to membrane with the amphiphatic helix a1 now lying along its surface. After oligomerisation to a dodecamer, helices a1 become inserted into the membrane forming a 130 Å hollow cylinder with a 30 Å aperture protruding through the membrane.

## 9. Synopsis and Medical Outlook

Neonatal sepsis is a syndrome caused by systemic inflammation and defined by clinical criteria such as tachycardia, respiratory distress, temperature instability, and unusually high amount of immature immune cells in the blood. Pathogenic bacteria that are aspirated by the fetus in the birth canal during parturition can cause neonatal sepsis if their infection is not controlled locally by the innate defence mechanisms of the respiratory and alveolar epithelia. Toxins, either proteinaceous or of other molecular nature, are important factors contributing to neonatal sepsis. While endotoxins such as the lipopolysaccharide (LPS) from Gram-negative bacteria contribute to the sepsis phenotype by activating monocytes and macrophages via Toll-like receptor 4 binding, pore-forming proteinaceous exotoxins act by permeabilising target membranes of host cells. Whereas the membrane targeting effects of PFTs, that is, engagement of membrane, oligomerisation, and pore formation, are well defined, their secondary downstream effects are manifold, owing to the fact that defined ionic and molecular gradients across cellular membranes modulate a diverse set of signalling cascades. Unregulated cell death and its consequences are important in neonatal sepsis [150]. The PFTs described above can cause direct necrotic cell death, which in the case of *E. coli*, GBS, and *S. aureus* contributes to overcoming the epithelial and endothelial barriers in the lung. LLO of *L. monocytogenes* is critical for cell invasion and cell-to-cell spread of this intracellular pathogen and thus also contributes to breaching the epithelial barriers of the host. Necrotic cell death can release proinflammatory cytokines from leukocytes thus contributing to the inflammatory storm during sepsis. Interestingly, several PFTs discussed above can induce the inflammasome: AFT [31], *β*-hemolysin/cytolysin [28], LLO [29, 151], HlyA [140], and leukocidins [32, 152]. Inflammasome activation ultimately may contribute to hyperinflammation in newborn infants similar to what has been shown in GBS and *E. coli* sepsis in mice [28, 153]. PFTs can also elicit and alter apoptosis of host immune cells, that is, AFT via caspase-2 [154, 155], leukocidins via activation of caspases 3 and 9 [156], LLO via release of cytochrome C from mitochondria [157], *β*-hemolysin/cytolysin independently of caspase activation [36, 39], HlyA [158], and ClyA [159], thus potentially contributing to the *apparent immunodeficiency* of the patient that is characteristic during stages of sepsis [160]. Despite the formation of hydrophilic channels by all PFTs, the ways in which they induce the inflammasome are varied, hinting towards the fact that functions of PFTs at cellular membranes are more subtle than might be expected from their general mode of action. In this respect, effects of sublytic concentrations of PFTs have recently been explored. For instance, sublytic doses of LLO induce mitochondrial network disorganisation with transient alteration of the metabolic state of the target cell, thus weakening the cell for *L. monocytogenes* entry without destroying it [161]. Moreover, targeting of organs by PFTs might also contribute to the septic phenotype. GBS *β*-hemolysin/cytolysin, for example, had marked effects on cardiomyocyte contractility and viability [162].

 Due to their role in neonatal sepsis and bacterial infection in general, PFTs present attractive therapeutic targets. In cases where membrane receptors have been defined, specific inhibitors, akin to viral entry inhibitors, might prevent membrane binding and pore formation. Monoclonal antibodies against PFTs that prevent membrane binding and/or refolding to the pore state could be a way of neutralising the toxin, at least in the blood stream. Moreover, vaccines based on PFTs may be used for immunizing women and thereby protecting newborn infants through placental transfer of specific immunoglobulins, given the fact that pneumolysin from *S. pneumoniae* is considered a vaccine candidate [163]. Indeed, novel vaccines based on AFT are currently developed [164]. As PFTs elicit specific cellular responses, it might, however, also be promising to designing therapeutics based on the pathways that the toxins induce in the target cell, as has been proposed for p38 MAPK and *β*-hemolysin/cytolysin from GBS [45]. In any case, it is exactly these cellular responses towards PFTs that need to be investigated in the context of neonatal sepsis in the future to improve therapeutic strategies.
